# Safety and Efficacy of Copanlisib in Combination with Nivolumab: A Phase Ib Study in Patients with Advanced Solid Tumors

**DOI:** 10.1158/2767-9764.CRC-24-0407

**Published:** 2025-03-14

**Authors:** Benedito A. Carneiro, Robert M. Jotte, Nashat Y. Gabrail, Kristopher Wentzel, Funan Huang, Shalini Chaturvedi, Anke Weispfenning, Florian Hiemeyer, Peter N. Morcos, Lidia Mongay Soler, Barrett H. Childs, Aaron R. Hansen

**Affiliations:** 1 Legorreta Cancer Center at Brown University, Lifespan Cancer Institute, Providence, Rhode Island.; 2 Rocky Mountain Cancer Centers, Denver, Colorado.; 3 US Oncology Research, Houston, Texas.; 4 Gabrail Cancer & Research Center, Canton, Ohio.; 5 The Angeles Clinic and Research Institute, A Cedars-Sinai Affiliate, Los Angeles, California.; 6 Bayer HealthCare Pharmaceuticals, Inc., Whippany, New Jersey.; 7 Pharmaceuticals Division, Bayer AG, Berlin, Germany.; 8 Princess Margaret Cancer Centre, Toronto, Canada.

## Abstract

**Purpose:**

Copanlisib in combination with immune checkpoint inhibitors demonstrated synergy and favorable antitumor immune responses in preclinical models. This study evaluated copanlisib plus nivolumab in adults with advanced solid tumors.

**Patients and Methods:**

In this phase Ib, nonrandomized, open-label, dose-escalation study, patients received intravenous nivolumab 240 mg (day 15 of cycle 1 and days 1 and 15 of subsequent cycles) plus intravenous copanlisib (45 or 60 mg on days 1, 8, and 15 of each cycle) in 28-day cycles. The primary objective was to determine the MTD and/or recommended phase II dose of copanlisib plus nivolumab. Secondary objectives were safety, tolerability, and efficacy. Exploratory objectives included evaluation of potentially predictive biomarkers.

**Results:**

Overall, 16 patients were treated [copanlisib: 45 mg (*n* = 5); 60 mg (*n* = 11)]. The most common cancer types at baseline were bladder (25.0%) and oropharyngeal (18.8%) cancers. No dose-limiting toxicities were observed; copanlisib 60 mg was deemed the recommended phase II dose in combination with nivolumab 240 mg. Grade 3 and 4 treatment-emergent adverse events were reported in 56.3% and 12.5% of patients, respectively; one grade 5 event was reported (unrelated to treatment). Overall, 18.8% of patients achieved a partial response. Evaluations of potential biomarkers did not correlate with response, but copanlisib-modulated biomarker changes were observed before nivolumab administration and were consistent and dose-dependent.

**Conclusions:**

No new safety concerns were identified with this combination, and preliminary efficacy indicated an antitumor effect. Data supported an immunomodulatory effect of copanlisib, suggesting that this combination may enhance the efficacy of immune checkpoint inhibitors.

**Significance:**

The combination of copanlisib and nivolumab was well tolerated and showed antitumor effects in patients with advanced solid tumors. The number of circulating myeloid-derived suppressive cells decreased 24 to 48 hours after treatment with copanlisib. Further investigation of copanlisib and nivolumab is warranted as a novel strategy to enhance the efficacy of checkpoint inhibitors.

## Introduction

The PI3K pathway is aberrantly activated in many cancer cells. It plays an important role in regulating immune responses, yielding a competitive cellular growth and survival advantage, and promoting metastases and resistance to conventional therapy (1). As such, the combination of PI3K inhibitors with immune checkpoint inhibitors (ICIs) has the potential to enhance antitumor response and improve patient outcomes.

Copanlisib is a pan–class I PI3K inhibitor with predominant activity against PI3K-α and -δ isoforms (2, 3), the most common isoforms in hematologic malignancies, which are also expressed in some solid tumors (4). Copanlisib was approved in the United States for adults with relapsed follicular lymphoma who have received at least two systemic therapies (5), following demonstration of its safety and efficacy in the open-label, single-arm, phase II CHRONOS-1 study (6); durable copanlisib efficacy and manageable safety have been reported in a long-term follow-up of the CHRONOS-1 study (7). More recently, copanlisib has been studied in combination with immunotherapy, both with rituximab (8) and chemoimmunotherapy (rituximab plus bendamustine; ref. 9), in patients with relapsed indolent non–Hodgkin lymphoma.

The binding of PD-1 ligands, PD-L1, and PD-L2 to PD-1 receptors inhibits T-cell proliferation and cytokine production and can suppress T-cell immune surveillance of tumors. Nivolumab is a human IgG4 mAb that binds to the PD-1 receptor and blocks its interaction with PD-1 ligands. It is approved for various advanced or metastatic solid tumor types, including non–small cell lung cancer, head and neck squamous cell carcinoma, microsatellite instability–high colorectal cancer, and hepatocellular carcinoma (10, 11). Despite its antitumor effects and ability to induce durable clinical benefit, ICI resistance induced by intratumoral oncogenic signaling and/or an immunosuppressive tumor microenvironment represents a significant therapeutic limitation (12).

The combination of copanlisib and anti–PD-1 or anti–PD-L1 has been studied in a panel of preclinical solid tumor models, in which they have demonstrated synergy and favorable antitumor immune responses (13). Pulsatile PI3K inhibition with copanlisib demonstrated immunomodulatory activity, and when combined with a PD-1 inhibitor, it led to complete tumor regression in ICI-resistant A20 lymphoma models and CT26 colorectal tumor models; furthermore, in a tumor rechallenge study conducted in the CT26 model 3 months after final treatment, no tumor growth was observed in mice that had received the combination of copanlisib and a PD-1 inhibitor, suggesting the generation of tumor-specific memory T cells (14). Based on these findings, the combination of copanlisib with anti–PD-1 or anti–PD-L1 represents a promising strategy to improve response to ICIs and/or overcome ICI resistance.

To characterize the potential of this combination in improving patient responses to ICIs, the data presented here include biomarker analyses, which sought to confirm the impact of dual PI3K and PD-1 inhibition on related signaling pathways, immune markers, and immune cell populations. This included assessments of proteins of interest that drive PI3K pathway signaling such as phosphorylated protein kinase B (pAKT) and phosphatase and tensin homolog (PTEN), evaluation of the immune composition of the tumor microenvironment via specific markers for regulatory T cells [Treg; cluster of differentiation (CD) 4 and forkhead box protein 3 (FoxP3)], infiltrating antitumor CD8 T cells (Ki-67 and CD8), and regulatory tumor-associated macrophages (TAMs; CD68 and PD-L1), and global flow cytometry analyses of immune cell subset populations before and after treatment.

Here, we report the results of the phase Ib portion of a phase Ib/II trial (NCT03735628), which aimed to determine the MTD and/or recommended phase II dose (RP2D), safety, biomarkers, preliminary efficacy, and pharmacokinetics (PK) of copanlisib in combination with nivolumab in patients with advanced solid tumors.

## Materials and Methods

### Study design and treatment

This open-label, multicenter trial (NCT03735628) of copanlisib plus nivolumab in patients with advanced solid tumors comprised a phase Ib dose-escalation study and a phase II dose-expansion study. The focus of this article is the phase Ib study, which was conducted at six sites across the United States and Canada. Representativeness of study participants is summarized in Supplementary Table S1.

The phase Ib study comprised screening, treatment, active follow-up, and long-term follow-up periods. Patients were not randomized but were assigned to cohorts with predefined dosing regimens.

Following screening, which occurred up to 28 days before the start of study treatment, two successive patient cohorts received treatment in 28-day cycles, with nivolumab at the standard dose of 240 mg and two increasing doses of copanlisib (45 or 60 mg). Nivolumab was administered at a fixed dose as an intravenous infusion on day 15 of cycle 1 and days 1 and 15 of subsequent cycles until disease progression, unacceptable toxicity, consent withdrawal, or up to 2 years, whichever came first. Nivolumab was provided as standard of care or by the study in accordance with local laws and requirements for each country where the study was conducted. Copanlisib was administered as a 1-hour intravenous infusion on days 1, 8, and 15 of each cycle until disease progression, unacceptable toxicity, consent withdrawal, or any other criteria for withdrawal were met.

Each cohort was to consist of at least four patients, and at least 13 patients evaluable for dose-limiting toxicities (DLT) were to be enrolled in the study overall; DLTs were defined as any of the prespecified events outlined in Supplementary Table S2 that occurred during cycle 1 or 2 and were considered related to the study treatment. Dose escalation, de-escalation, and determination of the MTD were guided through a modified toxicity probability interval (15), with the first dose-finding action performed after at least four patients evaluable for DLTs at a dose level were observed. The MTD, defined as the highest dose level that could be given so that the toxicity probability is <30%, was identified only when the following conditions were met: (i) at least six patients were treated at the identified dose level and at least 13 patients had been treated in the study; and (ii) the identified dose was recommended for patients based on statistical modeling and review of all clinical data by the study sponsor and investigators.

The active follow-up period comprised a mandatory clinical safety visit, which occurred 30 days (±5 days) after the last administration of study treatments. Patients who discontinued study treatment because of radiologic disease progression terminated the active follow-up after the clinical safety visit. Patients who discontinued study treatment for reasons other than death, consent withdrawal, or lost to follow-up continued with radiologic tumor assessments during the active follow-up period, which was terminated in those for whom radiologic disease progression, initiation of new anticancer treatment, or death were reported. Unless consent was withdrawn, patients who discontinued radiologic tumor assessments during active follow-up entered long-term follow-up for evaluation of overall survival (OS) up to 5 years after the last patient started study treatment.

This study was conducted in accordance with Good Clinical Practice guidelines, the ethical principles set forth in the Declaration of Helsinki and Council for International Organizations of Medical Sciences international ethical guidelines, and applicable laws and regulations. An institutional review board or independent ethics committee at each study site reviewed and approved the protocol used in the conduct of this study. All patients provided written, informed consent.

### Objectives

The primary objective was to estimate the MTD and/or RP2D of copanlisib in combination with nivolumab in patients with advanced solid tumors. Secondary objectives were to evaluate the safety and tolerability of copanlisib in combination with nivolumab and to characterize the PK and assess the efficacy of this drug combination. Exploratory objectives included the evaluation of the relationship between potentially predictive biomarkers and clinical outcomes.

### Patients

Eligibility criteria included patients, aged ≥18 years, with a histologically confirmed diagnosis of advanced solid tumors in which nivolumab is indicated (10); ≥1 measurable lesion (not previously irradiated) per RECIST version 1.1 (16); Eastern Cooperative Oncology Group performance status ≤1; life expectancy ≥12 weeks; and adequate bone marrow, liver, and kidney function. Exclusion criteria included active, known, or suspected autoimmune disease; previous exposure to copanlisib, nivolumab, or other ICIs; and known hypersensitivity to any of the study treatments, study drug classes, or excipients in the formulation.

### Assessments

Details on the assessment of DLTs are provided in the “Study design and treatment” section.

Safety analyses included assessment of the incidence, nature, and severity of adverse events (AEs) from the administration of study treatment until 30 days after the last administration of study treatment. AEs were reported according to Medical Dictionary for Regulatory Activities version 25.0 and graded using the NCI Common Terminology Criteria for Adverse Events version 5.0. Noninfectious pneumonitis was designated as an AE of special interest.

Blood samples for assessment of plasma copanlisib PK were collected on day 15 of cycle 1 (before infusion, 5–15 minutes after the start of infusion, end of infusion, and 1–5 hours after the start of infusion) and on day 15 of cycles 2 and 6 before infusion and end of infusion. Plasma samples were assayed using a validated LC/MS assay with a lower limit of quantification of 2 ng/mL. Blood samples for serum nivolumab PK were collected on day 15 of cycle 1 at 1 to 5 hours after the end of nivolumab infusion, and on day 15 of cycles 2 and 6 before infusion and 1 to 5 hours after the end of nivolumab infusion. Copanlisib and nivolumab PK were analyzed using previously established population PK models (17, 18). Model performance describing study PK was evaluated using prediction-corrected visual predictive checks. Copanlisib individual population PK model–predicted clearance and AUC from 0 to 168 hours at the nominal dose (AUC_[0–168]nd_) after the third 60 mg nominal dose in a sequence of three doses of 60 mg, each 1 week apart, were derived for individual patients. Nivolumab individual population PK model–predicted maximum concentration, average concentration, and minimum concentration following the first nivolumab dose were derived for individual patients.

Tumor response and progression were evaluated per RECIST version 1.1; tumor response per modified RECIST version 1.1 for immune-based therapeutics (iRECIST) was also documented as exploratory data for efficacy and treatment decisions. Tumor assessments were performed locally by study investigators using CT or MRI every 8 weeks (±7 days) starting from day 1 of cycle 1 until radiologic disease progression or end of the study; all scans were interpreted by the same investigator during the study, where possible. For patients who discontinued study treatment without radiologic disease progression during the treatment period, tumor assessments were continued every 8 weeks (±7 days) during the active follow-up period until progression of malignancy and/or start of subsequent anticancer treatment, whichever came first, or any other criterion for withdrawal was met. During the long-term follow-up period, patients were followed for OS every 3 months (±14 days) up to 5 years after the last patient started study treatment.

### Biomarker assessments

Blood samples for tumor biomarker analyses were collected at screening and at various timepoints: cycles 1 and 2; day 8 of cycles 3 and 4; day 8 of subsequent even cycles (cycle 6, cycle 8, etc.); and the end of treatment (Supplementary Table S3).

Fresh tumor biopsy was collected from consenting patients within 24 hours after treatment on day 8 of cycle 1 for the first 20 patients, day 8 of cycle 2 for the next 20 patients, and day 8 of cycle 1 for any patients beyond these first 40.

Biomarker evaluations were performed by Mosaic Laboratories, a College of American Pathologists/Clinical Laboratory Improvement Amendments–accredited laboratory, and included IHC testing of formalin-fixed, paraffin-embedded (FFPE) tissue samples from patients. FFPE human tissue controls, selected with positive and negative cell features, were provided by Mosaic Laboratories.

IHC testing was performed in accordance with the standard operating procedures and optimized/validated protocols of Mosaic Laboratories and included four single stains [pAKT on serine 473 (Ser473), pAKT on threonine 308 (Thr308), phosphorylated S6 ribosomal protein on serine 235/236 (pS6 ribosomal protein Ser235/236), and PTEN] and three multiplex stains (FoxP3 and CD4; Ki-67 and CD8; CD68, PD-L1, and CD3). For single-stain assays, IHC protein expression analyses were performed using anti–pAKT Ser473 (rabbit clone D9E, catalog #4060, RRID: AB_2315049), anti–pAKT Thr308 (rabbit clone C31E5E, catalog #2965, RRID: AB_2255933), anti–pS6 ribosomal protein Ser235/236 (rabbit clone D57.2.2E, catalog #4858, RRID: AB_916156), and anti-PTEN (rabbit clone 138G6, catalog #9559, RRID: AB_390810), all purchased from Cell Signaling Technology, Inc. For multiplex assays, IHC protein expression analyses were performed using anti-FoxP3 (mouse clone 236A/E7, catalog #ab20034, RRID: AB_445284), anti-CD4 (rabbit clone EPR6855, catalog #ab133616, RRID: AB_2750883), and anti–PD-L1 (rabbit clone SP142, catalog #AB228462, RRID: AB_2827816), purchased from Abcam; anti–Ki-67 (mouse clone MIB-1, catalog #M7240, RRID: AB_2142367), anti-CD8 (mouse clone C8/144B, catalog #M7103, RRID: AB_2075537), and anti-CD68 (mouse clone KP1, catalog #M0814, RRID: AB_2750584), purchased from Dako, an Agilent Technologies company; and anti-CD3 (mouse clone LN10, catalog #NCL-L-CD3-565, RRID: AB_563541), purchased from Leica Biosystems, Inc. Tissues were stained with Dako 3,3-diaminobenzidine (DAB; for single-stain assays); DAB and red and green chromogens (for CD68, PD-L1, and CD3 multiplex stains); or red and DAB chromogens (for other multiplex stains) and were centrally evaluated and scored by Mosaic Laboratories.

Flow cytometry in fresh whole blood, collected using Cyto-Chex (Alpha Laboratories) 5-mL tubes, was used to evaluate T cells, B cells, NK cells, monocytes [including myeloid-derived suppressive cells (MDSCs)], and various subtypes of immune markers. Assays were performed using eight-color flow cytometry and evaluated using BD FACSCanto software in real time at Covance Central Laboratory Services (RRID: SCR_018055). Additionally, whole blood for T-cell subset assays was collected in plastic sodium heparin 4-mL tubes.

Plasma cytokine (IFN-γ, IL-1β, IL-2, IL-4, IL-6, IL-8, IL-10, IL-12p70, IL-13, and TNFα) levels were measured by the study sponsor using the V-PLEX Proinflammatory Panel 1 (human) Kit from Meso Scale Discovery.

### Statistical analysis

Based on dosing simulations using a modified toxicity probability interval study design, 13 evaluable patients were deemed the minimum number of participants necessary for the dose evaluation. Assuming a rate of nonevaluable patients of 30%, it was estimated that 19 patients had to be recruited overall; however, recruitment continued until 13 evaluable patients were available for the final dose evaluation.

Statistical analyses were performed using SAS version 9.3 or higher (SAS Institute Inc.; RRID: SCR_008567), except for biomarker analyses, which were performed using R version 4.0.2 (The R Foundation for Statistical Computing; RRID: SCR_001905). Confirmatory analyses were not performed because of the exploratory nature of this study; all calculated confidence intervals (CI) were interpreted in an exploratory sense.

All patients who received at least 75% of the planned dose of each of the assigned study treatments, who received at least 50% of the planned combination doses administered together (i.e., on the same day), and who completed the scheduled safety assessments during the first two cycles and/or who experienced a DLT during the first two cycles were included in the MTD analysis set.

All patients who received at least one dose of study treatment were included in the full analysis set for safety and efficacy evaluations. Patients who did not achieve a best overall response of complete response (CR) or partial response (PR) or who had no postbaseline tumor assessment were considered nonresponders in efficacy analyses. Clopper–Pearson 95% CIs were generated for the objective response rate (ORR). Time-to-event endpoints were described using the Kaplan–Meier method; two-sided 95% CIs, based on the Greenwood formula, were generated for this estimate.

The Wilcoxon rank-sum test was used to compare the distribution of biomarkers at baseline between groups of patients based on best overall response [CR, PR, stable disease (SD) vs. progressive disease (PD), not evaluable, or unknown].

### Data availability

Availability of the data underlying this publication will be determined according to Bayer’s commitment to the European Federation of Pharmaceutical Industries and Associations/Pharmaceutical Research and Manufacturers of America “Principles for responsible clinical trial data sharing.” This pertains to scope, timepoint, and process of data access.

As such, Bayer commits to sharing, upon request from qualified scientific and medical researchers, patient-level clinical trial data, study-level clinical trial data, and protocols from clinical trials in patients for medicines and indications approved in the United States and European Union, as necessary for conducting legitimate research. This applies to data on new medicines and indications that have been approved by the EU and US regulatory agencies on or after January 1, 2014.

Interested researchers can use www.vivli.org to request access to anonymized patient-level data and supporting documents from clinical studies in order to conduct further research. Information on the Bayer criteria for listing studies and other relevant information is provided in the member section of the portal.

Data access will be granted to anonymized patient-level data, protocols, and clinical study reports after approval by an independent scientific review panel. Bayer is not involved in the decisions made by the independent review panel. Bayer will take all necessary measures to ensure that patient privacy is safeguarded.

## Results

### Patients

Between October 17, 2018, and October 13, 2022, 16 patients were enrolled in the study and received at least one dose of study treatment (full analysis set). Of these patients, five received copanlisib 45 mg plus nivolumab 240 mg (copanlisib 45-mg cohort) and 11 received copanlisib 60 mg plus nivolumab 240 mg (copanlisib 60-mg cohort). All patients in the full analysis set received at least one dose of copanlisib, and 15 patients received at least one dose of nivolumab. Overall, the median age of patients was 65.0 years, and 12 patients (75.0%) were male (Table 1). The most common cancer type was bladder cancer (*n* = 4, 25.0%), followed by oropharyngeal cancer (*n* = 3, 18.8%). All patients had stage IV cancer at study entry (*n* = 16, 100%), and most had received one line of systemic anticancer therapy (*n* = 12, 75.0%), with a median time since most recent progression of approximately 3.5 weeks. No patients had previously received copanlisib or ICI treatment.

**Table 1:** Patient demographics and baseline cancer characteristics

Demographic or characteristic	Copanlisib 45 mg plus nivolumab 240 mg	Copanlisib 60 mg plus nivolumab 240 mg	Total
	(*n* = 5)	(*n* = 11)	(*N* = 16)
Male, *n* (%)	4 (80.0)	8 (72.7)	12 (75.0)
Median age, years (range)	64.0 (58–75)	66.0 (37–89)	65.0 (37–89)
Cancer type, *n* (%)			
Bladder	0	4 (36.4)	4 (25.0)
Choroidal melanoma	1 (20.0)	0	1 (6.3)
Head and neck	0	1 (9.1)	1 (6.3)
Kidney	0	1 (9.1)	1 (6.3)
Non–small cell lung	0	1 (9.1)	1 (6.3)
Oropharyngeal	2 (40.0)	1 (9.1)	3 (18.8)
Small cell lung	2 (40.0)	0	2 (12.5)
Squamous cell carcinoma	0	2 (18.2)	2 (12.5)
Squamous cell carcinoma of the tongue	0	1 (9.1)	1 (6.3)
Median time from initial diagnosis, months (range)	13.0 (10.0–67.9)	11.1 (4.4–53.8)	12.1 (4.4–67.9)
Baseline ECOG PS score, *n* (%)			
0	2 (40.0)	4 (36.4)	6 (37.5)
1	3 (60.0)	7 (63.6)	10 (62.5)
Disease stage at study entry, *n* (%)			
IV	5 (100)	10 (90.9)	15 (93.8)
IV B	0	1 (9.1)	1 (6.3)
Previous lines of systemic therapy, *n* (%)			
None	1 (20.0)	1 (9.1)	2 (12.5)
1	3 (60.0)	9 (81.8)	12 (75.0)
2	0	1 (9.1)	1 (6.3)
4	1 (20.0)	0	1 (6.3)
Time since most recent progression, months (range)	1.5 (0.3–5.9)	0.6 (0.0–8.7)	0.8 (0.0–8.7)

Abbreviation: ECOG PS, Eastern Cooperative Oncology Group performance status.

At database cutoff (November 8, 2022), no patients were receiving treatment; the most common reason for discontinuation was radiologic disease progression (*n* = 7, 43.8%). Patients received up to 46 cycles in the copanlisib 45-mg cohort and up to 20 cycles in the copanlisib 60-mg cohort.

### RP2D

Fourteen patients were evaluable for DLTs (four in the copanlisib 45-mg cohort and 10 in the copanlisib 60-mg cohort). No DLTs were observed, and copanlisib 60 mg was determined as the RP2D to be used in combination with nivolumab 240 mg in patients with solid tumors.

### Safety

Overall, 16 patients (100%) experienced a treatment-emergent AE (TEAE), nine (56.3%) and two (12.5%) of whom reported grade 3 and grade 4 TEAEs, respectively. The most common any-grade TEAEs were constipation and fatigue (both *n* = 8, 50.0%), hypertension and diarrhea (both *n* = 7, 43.8%), and nausea and maculopapular rash (both *n* = 6, 37.5%; Table 2). In total, concomitant topical and systemic corticosteroids were each used by seven patients (43.8%).

**Table 2:** Summary of patient safety outcomes (full analysis set)

*n* (%)	Copanlisib 45 mg plus nivolumab 240 mg (*n* = 5)						Copanlisib 60 mg plus nivolumab 240 mg (*n* = 11)						Total (*N* = 16)					
	Grade 1	Grade 2	Grade 3	Grade 4	Grade 5	Any grade	Grade 1	Grade 2	Grade 3	Grade 4	Grade 5	Any grade	Grade 1	Grade 2	Grade 3	Grade 4	Grade 5	Any grade
TEAEs occurring in ≥10% of patients overall																		
Constipation	2 (40.0)	0	0	0	0	2 (40.0)	4 (36.4)	2 (18.2)	0	0	0	6 (54.5)	6 (37.5)	2 (12.5)	0	0	0	8 (50.0)
Fatigue	0	4 (80.0)	0	0	0	4 (80.0)	2 (18.2)	2 (18.2)	0	0	0	4 (36.4)	2 (12.5)	6 (37.5)	0	0	0	8 (50.0)
Diarrhea	2 (40.0)	1 (20.0)	0	0	0	3 (60.0)	2 (18.2)	2 (18.2)	0	0	0	4 (36.4)	4 (25.0)	3 (18.8)	0	0	0	7 (43.8)
Hypertension	0	1 (20.0)	3 (60.0)	0	0	4 (80.0)	0	0	3 (27.3)	0	0	3 (27.3)	0	1 (6.3)	6 (37.5)	0	0	7 (43.8)
Nausea	3 (60.0)	0	0	0	0	3 (60.0)	1 (9.1)	2 (18.2)	0	0	0	3 (27.3)	4 (25.0)	2 (12.5)	0	0	0	6 (37.5)
Maculopapular rash	1 (20.0)	2 (40.0)	0	0	0	3 (60.0)	1 (9.1)	1 (9.1)	1 (9.1)	0	0	3 (27.3)	2 (12.5)	3 (18.8)	1 (6.3)	0	0	6 (37.5)
Back pain	2 (40.0)	0	0	0	0	2 (40.0)	2 (18.2)	0	1 (9.1)	0	0	3 (27.3)	4 (25.0)	0	1 (6.3)	0	0	5 (31.3)
Increased blood creatinine	1 (20.0)	0	0	0	0	1 (20.0)	4 (36.4)	0	0	0	0	4 (36.4)	5 (31.3)	0	0	0	0	5 (31.3)
Cough	2 (40.0)	2 (40.0)	0	0	0	4 (80.0)	1 (9.1)	0	0	0	0	1 (9.1)	3 (18.8)	2 (12.5)	0	0	0	5 (31.3)
Dizziness	3 (60.0)	0	0	0	0	3 (60.0)	2 (18.2)	0	0	0	0	2 (18.2)	5 (31.3)	0	0	0	0	5 (31.3)
Hypokalemia	2 (40.0)	0	1 (20.0)	0	0	3 (60.0)	1 (9.1)	0	1 (9.1)	0	0	2 (18.2)	3 (18.8)	0	2 (12.5)	0	0	5 (31.3)
Hypotension	1 (20.0)	1 (20.0)	0	0	0	2 (40.0)	2 (18.2)	0	1 (9.1)	0	0	3 (27.3)	3 (18.8)	1 (6.3)	1 (6.3)	0	0	5 (31.3)
Pruritus	0	1 (20.0)	0	0	0	1 (20.0)	1 (9.1)	2 (18.2)	1 (9.1)	0	0	4 (36.4)	1 (6.3)	3 (18.8)	1 (6.3)	0	0	5 (31.3)
Stomatitis	2 (40.0)	0	0	0	0	2 (40.0)	2 (18.2)	1 (9.1)	0	0	0	3 (27.3)	4 (25.0)	1 (6.3)	0	0	0	5 (31.3)
Decreased weight	1 (20.0)	0	1 (20.0)	0	0	2 (40.0)	3 (27.3)	0	0	0	0	3 (27.3)	4 (25.0)	0	1 (6.3)	0	0	5 (31.3)
Arthralgia	2 (40.0)	0	0	0	0	2 (40.0)	1 (9.1)	1 (9.1)	0	0	0	2 (18.2)	3 (18.8)	1 (6.3)	0	0	0	4 (25.0)
Dyspnea	1 (20.0)	1 (20.0)	0	0	0	2 (40.0)	2 (18.2)	0	0	0	0	2 (18.2)	3 (18.8)	1 (6.3)	0	0	0	4 (25.0)
Pyrexia	0	1 (20.0)	1 (20.0)	0	0	2 (40.0)	1 (9.1)	0	1 (9.1)	0	0	2 (18.2)	1 (6.3)	1 (6.3)	2 (12.5)	0	0	4 (25.0)
Upper abdominal pain	3 (60.0)	0	0	0	0	3 (60.0)	0	0	0	0	0	0	3 (18.8)	0	0	0	0	3 (18.8)
Increased alanine aminotransferase	0	1 (20.0)	0	0	0	1 (20.0)	2 (18.2)	0	0	0	0	2 (18.2)	2 (12.5)	1 (6.3)	0	0	0	3 (18.8)
Anemia	1 (20.0)	0	1 (20.0)	0	0	2 (40.0)	1 (9.1)	0	0	0	0	1 (9.1)	2 (12.5)	0	1 (6.3)	0	0	3 (18.8)
Candida infection	0	1 (20.0)	0	0	0	1 (20.0)	1 (9.1)	1 (9.1)	0	0	0	2 (18.2)	1 (6.3)	2 (12.5)	0	0	0	3 (18.8)
Decreased appetite	0	1 (20.0)	0	0	0	1 (20.0)	1 (9.1)	1 (9.1)	0	0	0	2 (18.2)	1 (6.3)	2 (12.5)	0	0	0	3 (18.8)
Flank pain	1 (20.0)	0	0	0	0	1 (20.0)	1 (9.1)	0	1 (9.1)	0	0	2 (18.2)	2 (12.5)	0	1 (6.3)	0	0	3 (18.8)
Headache	0	0	0	0	0	0	3 (27.3)	0	0	0	0	3 (27.3)	3 (18.8)	0	0	0	0	3 (18.8)
Hematuria	0	1 (20.0)	0	0	0	1 (20.0)	0	1 (9.1)	1 (9.1)	0	0	2 (18.2)	0	2 (12.5)	1 (6.3)	0	0	3 (18.8)
Hyperglycemia	1 (20.0)	0	0	0	0	1 (20.0)	0	2 (18.2)	0	0	0	2 (18.2)	1 (6.3)	2 (12.5)	0	0	0	3 (18.8)
Hypomagnesemia	1 (20.0)	0	0	1 (20.0)	0	2 (40.0)	1 (9.1)	0	0	0	0	1 (9.1)	2 (12.5)	0	0	1 (6.3)	0	3 (18.8)
Myalgia	1 (20.0)	1 (20.0)	0	0	0	2 (40.0)	0	1 (9.1)	0	0	0	1 (9.1)	1 (6.3)	2 (12.5)	0	0	0	3 (18.8)
Neutropenia	0	2 (40.0)	1 (20.0)	0	0	3 (60.0)	0	0	0	0	0	0	0	2 (12.5)	1 (6.3)	0	0	3 (18.8)
Pain in extremities	0	1 (20.0)	0	0	0	1 (20.0)	1 (9.1)	1 (9.1)	0	0	0	2 (18.2)	1 (6.3)	2 (12.5)	0	0	0	3 (18.8)
Rash	1 (20.0)	0	0	0	0	1 (20.0)	1 (9.1)	0	1 (9.1)	0	0	2 (18.2)	2 (12.5)	0	1 (6.3)	0	0	3 (18.8)
Urinary tract infection	0	0	0	0	0	0	0	2 (18.2)	1 (9.1)	0	0	3 (27.3)	0	2 (12.5)	1 (6.3)	0	0	3 (18.8)
Copanlisib-related TEAEs occurring in ≥10% of patients overall																		
Neutropenia	0	2 (40.0)	1 (20.0)	0	0	3 (60.0)	0	0	0	0	0	0	0	2 (12.5)	1 (6.3)	0	0	3 (18.8)
Thrombocytopenia	1 (20.0)	0	1 (20.0)	0	0	2 (40.0)	0	0	0	0	0	0	1 (6.3)	0	1 (6.3)	0	0	2 (12.5)
Diarrhea	1 (20.0)	1 (20.0)	0	0	0	2 (40.0)	0	1 (9.1)	0	0	0	1 (9.1)	1 (6.3)	2 (12.5)	0	0	0	3 (18.8)
Nausea	1 (20.0)	0	0	0	0	1 (20.0)	0	2 (18.2)	0	0	0	2 (18.2)	1 (6.3)	2 (12.5)	0	0	0	3 (18.8)
Stomatitis	1 (20.0)	0	0	0	0	1 (20.0)	2 (18.2)	1 (9.1)	0	0	0	3 (27.3)	3 (18.8)	1 (6.3)	0	0	0	4 (25.0)
Fatigue	0	3 (60.0)	0	0	0	3 (60.0)	2 (18.2)	1 (9.1)	0	0	0	3 (27.3)	2 (12.5)	4 (25.0)	0	0	0	6 (37.5)
Decreased platelet count	1 (20.0)	0	0	0	0	1 (20.0)	1 (9.1)	0	0	0	0	1 (9.1)	2 (12.5)	0	0	0	0	2 (12.5)
Decreased weight	0	0	1 (20.0)	0	0	1 (20.0)	1 (9.1)	0	0	0	0	1 (9.1)	1 (6.3)	0	1 (6.3)	0	0	2 (12.5)
Hyperglycemia	1 (20.0)	0	0	0	0	1 (20.0)	0	2 (18.2)	0	0	0	2 (18.2)	1 (6.3)	2 (12.5)	0	0	0	3 (18.8)
Arthralgia	1 (20.0)	0	0	0	0	1 (20.0)	1 (9.1)	0	0	0	0	1 (9.1)	2 (12.5)	0	0	0	0	2 (12.5)
Maculopapular rash	0	1 (20.0)	0	0	0	1 (20.0)	1 (9.1)	0	0	0	0	1 (9.1)	1 (6.3)	1 (6.3)	0	0	0	2 (12.5)
Hypertension	0	1 (20.0)	2 (40.0)	0	0	3 (60.0)	0	0	3 (27.3)	0	0	3 (27.3)	0	1 (6.3)	5 (31.3)	0	0	6 (37.5)

Overall, immune-related TEAEs were low [pneumonitis (grade 1, *n* = 1, 6.3%), colitis (grade 1, *n* = 1, 6.3%), hypothyroidism (grade 2, *n* = 1, 6.3%), rash (grade 1, *n* = 2, 12.5%), and maculopapular rash (grade 1, *n* = 2, 12.5%; grade 2, *n* = 3, 18.8%; grade 3, *n* = 1, 6.3%)].

Fourteen patients (87.5%) reported at least one copanlisib-related TEAE overall. The most common any-grade copanlisib-related TEAEs were fatigue (*n* = 6, 37.5%), hypertension (*n* = 6, 37.5%), stomatitis (*n* = 4, 25.0%), nausea (*n* = 3, 18.8%), and hyperglycemia (*n* = 3, 18.8%). Seven patients (43.8%) reported a grade 3 copanlisib-related TEAE, four (25.0%) reported a grade 3 nivolumab-related TEAE, and there were no reports of grade 4 or 5 drug-related TEAEs. Grade 3 copanlisib-related TEAEs by preferred term were hypertension (*n* = 2, 40.0%) and neutropenia, thrombocytopenia, increased lipase, and decreased weight (*n* = 1, 20.0%) in one patient each in the copanlisib 45-mg cohort and hypertension (*n* = 3, 27.3%) and rash (*n* = 1, 9.1%) in the copanlisib 60-mg cohort. Grade 3 nivolumab-related TEAEs by preferred term were increased lipase (*n* = 1, 20.0%, resolved) and decreased weight (*n* = 1, 20.0%) in the copanlisib 45-mg cohort and pruritus (*n* = 1, 9.1%) and maculopapular rash (*n* = 1, 9.1%) in the copanlisib 60-mg cohort; grade 3 decreased weight did not resolve, and grade 3 pruritus and maculopapular rash did not resolve but lessened in severity without dose modification.

Overall, serious TEAEs were reported in seven patients (43.8%; two in the copanlisib 45-mg cohort and five in the copanlisib 60-mg cohort). In the copanlisib 45-mg cohort, general physical health deterioration (grade 5) was reported in one patient and pyrexia and pneumonia (both grade 3) in another patient, which were all considered unrelated to study treatments. In the copanlisib 60-mg cohort, hypertension (grade 3; considered copanlisib-related), bacterial meningitis (grade 4), back pain (grade 3), pyrexia (grade 3), and hematuria (grade 3) were reported in one patient (9.1%) each.

TEAEs leading to discontinuation of copanlisib (*n* = 3, 18.8%) were all serious TEAEs and occurred only in the copanlisib 60-mg cohort [bacterial meningitis (grade 4), back pain (grade 2), and hematuria (grade 3)]. TEAEs leading to copanlisib dose reduction or copanlisib interruption were reported in three (18.8%) and five (31.3%) patients overall, respectively.

One patient (6.3%), who was in the copanlisib 45-mg cohort, experienced an AE of special interest of noninfectious pneumonitis (grade 1) during the study.

Ten patients (62.5%) died during the study (two in the copanlisib 45-mg cohort and eight in the 60-mg cohort). The causes of death were general physical health deterioration (*n* = 2) in the copanlisib 45-mg cohort and PD (*n* = 5) and unknown cause (*n* = 3) in the copanlisib 60-mg cohort. No AE leading to death was deemed to be related to copanlisib or nivolumab.

### PK

A total of 16 and 15 patients had valid postbaseline copanlisib and nivolumab PK observations, respectively, and were included in PK analyses. The previously established copanlisib population PK models adequately captured the available copanlisib PK across the two dose levels (Supplementary Fig. S1). Application of the established nivolumab PK model led to some bias, and the model was successfully applied after re-estimating all random effects using data from this study (Supplementary Fig. S2). Individual copanlisib clearance values in this study were generally consistent with historical data from the CHRONOS-1 study (Supplementary Fig. S3; ref. 19). The individual copanlisib AUC_[0-168]nd_ values confirmed dose-dependent PK across the two dose levels, with no obvious influence of copanlisib exposure on tumor response (Supplementary Fig. S4). Nivolumab geometric mean (coefficient of variation) population PK model–predicted maximum, average, and minimum concentrations were 65.7 μg/mL (30.4%), 29.5 μg/mL (18.7%), and 18.9 μg/mL (20.0%), respectively.

### Efficacy

**Figure 1:**
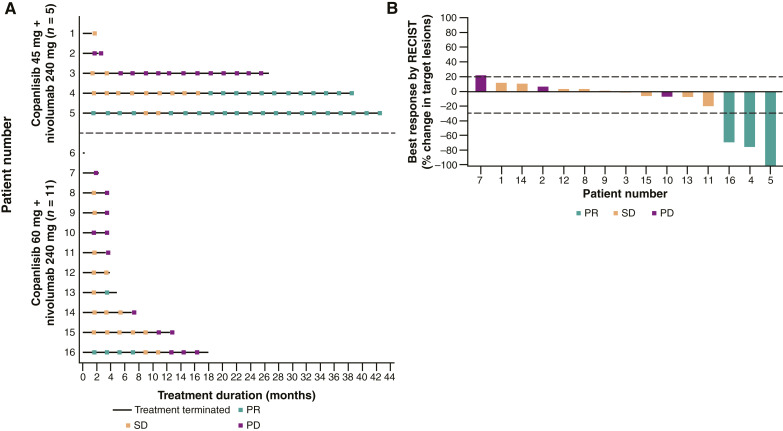
Response and duration of treatment (**A**) and percentage change in target lesions (**B**; full analysis set).

Three patients (18.8%) achieved a confirmed PR (copanlisib 45-mg cohort: *n* = 2, ORR = 40.0%; copanlisib 60-mg cohort: *n* = 1, ORR = 9.1%) per RECIST version 1.1 and a >60% decrease in their target lesions (Table 3; Fig. 1A and B); the tumor types for these three patients were urothelial carcinoma of the bladder, choroidal melanoma, and oropharyngeal tongue cancer. Nine patients (56.3%) achieved a best overall response of SD, and the disease control rate was 75.0% (*n* = 12). Three patients (18.8%) had PD.

**Table 3:** Best overall response per RECIST version 1.1 (full analysis set)

Response	Copanlisib 45 mg plus nivolumab 240 mg	Copanlisib 60 mg plus nivolumab 240 mg	Total
	(*n* = 5)	(*n* = 11)	(*N* = 16)
Confirmed best overall response, *n* (%)^a^			
CR	0	0	0
PR	2 (40.0)	1 (9.1)	3 (18.8)
SD	2 (40.0)	7 (63.6)	9 (56.3)
PD	1 (20.0)	2 (18.2)	3 (18.8)
No tumor assessment	0	1 (9.1)	1 (6.3)
ORR, *n* (%)^b^	2 (40.0)	1 (9.1)	3 (18.8)
95% CI^c^	5.3, 85.3	0.2, 41.3	4.0, 45.6
DCR, *n* (%)^d^	4 (80.0)	8 (72.7)	12 (75.0)
95% CI^c^	28.4, 99.5	39.0, 94.0	47.6, 92.7

Abbreviation: DCR, disease control rate.

a Confirmation was required to be obtained at a consecutive assessment at least 4 weeks later for objective responses of CR or PR.

b ORR was defined as patients with a best overall response of CR or PR.

c Based on two-sided Clopper–Pearson CI.

d DCR was defined as patients with a best overall response of CR, PR, or SD.

The median time to response was 9.9 months (range, 1.5–18.3) for the two patients in the copanlisib 45-mg cohort and 1.7 months (range not evaluable) for the one patient in the copanlisib 60-mg cohort, who had a PR. The median duration of response was not estimable overall. The median duration of SD was 3.5 months (range, 1.6–14.1) overall [copanlisib 45-mg cohort: 2.8 months (range, 1.7–5.4); copanlisib 60-mg cohort: 3.5 months (range, 1.6–14.1)].

**Figure 2:**
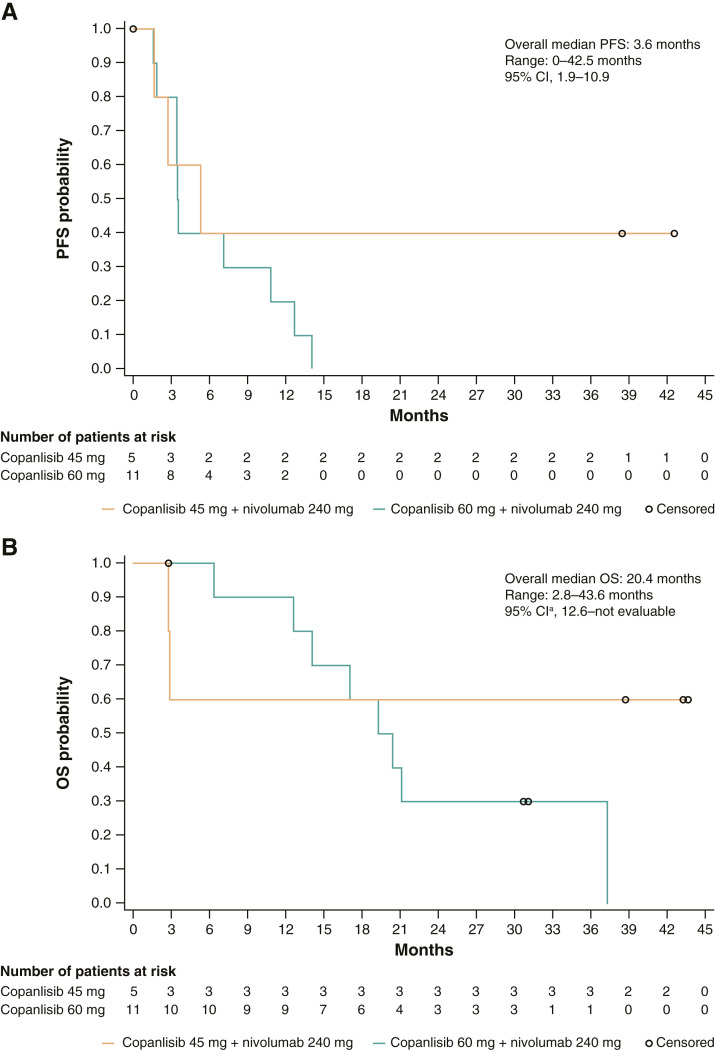
Secondary efficacy endpoints. Data are shown for progression-free survival (**A**) and OS (**B**) as assessed locally by study investigators (full analysis set). ^a^95% CI based on the Greenwood formula. PFS, progression-free survival.

Overall, the median progression-free survival was 3.6 months (range, 0–42.5; Fig. 2A). Numerically longer median progression-free survival was observed in the copanlisib 45-mg cohort versus the copanlisib 60-mg cohort [5.4 months (range, 1.7–42.5) vs. 3.5 months (range, 0–14.1)]. The median time to progression was 3.6 months (range, 0–42.5) overall. The median OS was 20.4 months (range, 2.8–43.6) overall (Fig. 2B). The median OS was not estimable in the copanlisib 45-mg cohort and was 19.8 months (range, 2.8–37.3) in the copanlisib 60-mg cohort. Kaplan–Meier survival analyses generally showed an earlier occurrence of death or progression and later censoring in the copanlisib 45-mg cohort versus the copanlisib 60-mg cohort.

### ORR per iRECIST

The confirmed ORR per iRECIST was 18.8% (*n* = 3) overall (copanlisib 45-mg cohort: *n* = 2, ORR = 40.0%; copanlisib 60-mg cohort: *n* = 1, ORR = 9.1%); the two patients in the copanlisib 45-mg cohort achieved a confirmed immune PR, and the patient in the copanlisib 60-mg cohort achieved a confirmed immune CR.

### Biomarkers

Given the central role of PI3K activation in promoting tumor growth and survival (20), the AKT pathway was evaluated via the assessment of pAKT levels using IHC. FFPE tissue samples from 15 patients (four from the 45-mg cohort and 11 from the 60-mg cohort) underwent IHC testing at baseline. Expression of these IHC markers was then correlated with observed patient efficacy and categorization in the responder group (confirmed response, PR, or SD) or the nonresponder group (PD, not evaluable, or unknown). In total, pAKT Ser473, pAKT Thr308, and pS6 ribosomal protein Ser235/236 single staining was performed on samples from nine patients, with median positive expression levels of 0%, 2%, and 2%, respectively. There was no significant association between expression of these biomarkers and treatment response (*P* = 0.220, *P* = 0.223, and *P* = 0.091 for six responders and three nonresponders). As it has been hypothesized that the tumor suppressor PTEN is the main node in the PI3K–AKT inhibitory network (21), patients with PTEN loss may demonstrate high PI3K pathway activation, which may be inhibited effectively by copanlisib. PTEN staining was performed on 14 FFPE samples from 14 patients (association with response *P* = 0.942 for 10 responders and four nonresponders), five samples (35.7%) of which demonstrated complete PTEN loss or a positive expression level of 0%. There was no significant correlation between PTEN loss and treatment response. Additionally, tumor infiltration of Tregs and M2-subtype TAMs (13) was investigated using IHC to assess the immunomodulatory effects of the combination of copanlisib and nivolumab. FoxP3 and CD4 multiplex staining was performed on 12 samples; the median expression level of CD4^+^ cells that were FoxP3^+^ was 3.95%, confirming low Treg presence (association with response *P* = 0.196 for nine responders and three nonresponders). High CD8^+^ T-cell densities are usually found to be associated with favorable prognosis in solid tumors (22). However, Ki-67^+^ CD8^+^ T cells have been shown to represent an activated and antitumor-specific subset of CD8^+^ T cells (23). Therefore, Ki-67 and CD8 multiplex staining was performed on FFPE samples from 14 patients. A median expression level (i.e., the percentage of CD8^+^ cells that are Ki-67^+^) of 16.25% was found, suggesting a highly activated CD8^+^ antitumor subset. However, there was no significant association between Ki-67 expression and tumor response (*P* = 0.832 for 10 responders and four nonresponders). CD68 (a pan-macrophage marker) and PD-L1 (a transmembrane co-inhibitory factor of immune response) were also evaluated. The median expression levels of CD68^+^, PD-L1^+^ (H-score), and CD68^+^ PD-L1^+^ were 1.87% (*n* = 15, association with response *P* = 0.396 for 11 responders and four nonresponders), 9.12% (*n* = 15, *P* = 0.648 for 11 responders and four nonresponders), and 37.17% (*n* = 12, *P* = 1.00 for nine responders and three nonresponders), respectively. This confirmed low levels of macrophages positive for inhibitory PD-L1, which could have impaired positive treatment responses. Overall, the presence of biomarkers, ranging from pAKT and PTEN as pathway markers, Tregs, CD8^+^ cells, and PD-L1 as immune markers, and Ki-67 as a proliferation marker, was tested using IHC on baseline specimens in our study. However, none of the markers tested correlated with treatment response, likely due to the limited number of samples and minimal overall efficacy observed in the study.

To better understand the composition of immune cell types before and after copanlisib treatment, peripheral blood samples were collected, and flow cytometry was performed on several immune cell subsets, including various T-cell phenotypes (CD4^+^ and CD8^+^), Treg polarization subsets (central memory to effector memory cells), B cells, NK cells, monocytes, and MDSCs. The aim of these analyses was to measure changes in immune cell populations following copanlisib treatment and identify immune cell subsets that are predictive of response.

**Figure 3:**
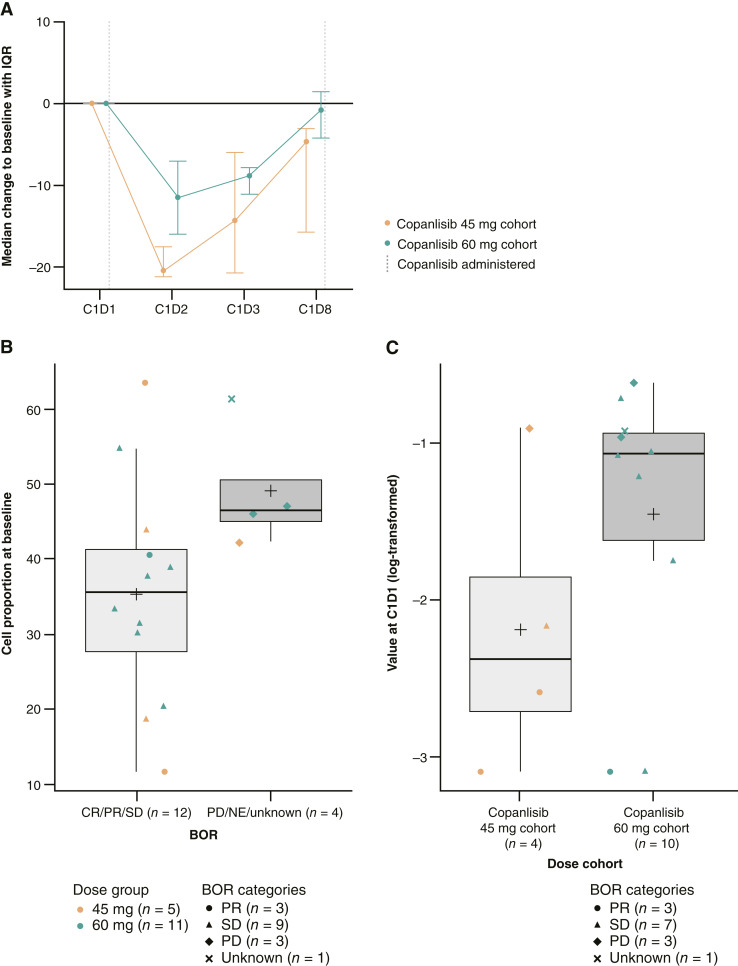
Exploratory biomarker endpoints. Data are shown for change from baseline in the median proportion of MDSCs over time (**A**), percentage of CD45RA^−^ (CD3^+^/CD8^+^) cells and clearance at baseline by BOR (**B**), and circulating IL-2 levels at baseline by BOR (**C**). BOR, best overall response; C, cycle; D, day; NE, not evaluable.

The most consistent changes after treatment across both datasets were seen in MDSCs, in which a copanlisib-modulated decrease in CD14^+^ MDSCs was observed by days 2 and 3 of cycle 1, before nivolumab administration. This change was consistent with both doses of copanlisib and was transient in nature (returned to baseline levels by day 8 of cycle 1). The monocytic MDSCs, characterized by CD14^+^ DR^lo^ CD11b^+^ CD33^+^ Lin^−^ gated on mononuclear cells, decreased significantly with both doses of copanlisib by a median of approximately 10% to 20% from baseline at day 2 of cycle 1 (Fig. 3A).

Additionally, baseline levels of cell types from flow cytometry and circulating cytokine levels as assessed via immunoassay were evaluated for association with clinical response. A lower level of CD8^+^ effector memory cells, defined by the percentage of CD45RA^−^/CCR7^−^ (CD3^+^/CD8^+^) cells at baseline, seemed to correlate with a greater patient response [*P* = 0.060 (unadjusted); *P* = 0.768 (adjusted); Fig. 3B]. In addition, lower baseline levels of IL-2, a cytokine known to regulate aspects of T-cell biology, were associated with a greater overall patient response [*P* = 0.019 (unadjusted); Fig. 3C].

## Discussion

To the best of our knowledge, this article reports the results of the first study investigating the safety and feasibility of administering the combination of copanlisib and nivolumab in patients with advanced solid tumors. In the phase Ib dose-escalation portion described in this study, no DLTs occurred during the first two treatment cycles, and copanlisib 60 mg in combination with nivolumab 240 mg was established as the RP2D, consistent with the full dose of copanlisib approved for the treatment of adults with relapsed follicular lymphoma (5). Upon completion of the phase Ib study, the phase II part was canceled because of strategic decisions by the trial sponsor.^1^

Copanlisib and nivolumab have well-characterized safety profiles as monotherapies, with that of copanlisib characterized by hyperglycemia, hypertension, and neutropenia that are transient and manageable in nature and that of nivolumab characterized by immune-related AEs that are uncommon with copanlisib (6, 25). Overall, TEAEs reported herein were generally consistent with those reported with copanlisib and nivolumab administered as monotherapy (6, 26–28), with no new safety signals reported for these therapies in combination. The most common TEAEs overall were constipation, fatigue, diarrhea, hypertension, nausea, and maculopapular rash. Hypertension was reported at a frequency of 43.8% overall, consistent with the frequency reported in copanlisib monotherapy studies in patients with relapsed or refractory diffuse large B-cell lymphoma (40.3%) and relapsed or refractory indolent lymphoma (30%); by contrast, hyperglycemia and neutropenia were both reported in 18.8% of patients overall, which is similar to or lower than that reported in copanlisib monotherapy studies, in which the frequency of these events ranged from 32.8% to 59.5% and from 13.0% to 30.0%, respectively (6, 19, 26, 29). Although the higher frequency of hypertension reported here is consistent across previous clinical trials of copanlisib (8, 30), the mechanism of copanlisib-induced hypertension is still not well understood. However, potential explanations for this toxicity include copanlisib-induced endothelial dysfunction and acute vasoconstriction following intravenous infusion (26, 31, 32).

Given the mechanism of action of ICIs, which promote the activation and proliferation of T cells, and the ability of T cells to infiltrate most organs, ICIs can induce a range of immune-related AEs (33). Common immune-related AEs reported with PD-1 inhibitors include fatigue, rash, and diarrhea (33), the frequency of which in the present study was similar to or higher than that observed in nivolumab monotherapy studies (27, 28). Moreover, the frequency of these events was generally similar to interim results in an ongoing combination study of nivolumab and copanlisib in patients with Richter transformation or transformed non–Hodgkin lymphoma (34) and in combination studies of other PI3K and PD-1 inhibitors in patients with metastatic castration-resistant prostate cancer (35).

Serious TEAEs were reported in approximately 40% of patients in each treatment cohort, most of which were grade 3 in severity. Grade 3 copanlisib-related TEAEs were approximately twofold higher in the copanlisib 45-mg cohort than in the copanlisib 60-mg cohort; however, patient numbers were small in both cohorts. There were no grade 4 or 5 copanlisib-related TEAEs.

Both copanlisib and nivolumab are associated with pneumonitis (6, 25), which can be severe or even life-threatening (36, 37); therefore, this was an AE of special interest in the present study and was closely monitored. The etiology of pneumonitis driven by these drug classes remains unclear but is presumed to be related to ICI-mediated immune dysregulation or an inflammatory-related sequela of PI3K inhibition (38, 39). The rate of pneumonitis reported herein (6.3%) was generally consistent with that reported in copanlisib (4.5%–8.0%) and nivolumab (2.0%–6.0%) monotherapy trials (6, 26–28), suggesting acceptable tolerability of copanlisib in combination with nivolumab.

The small sample size seen here provides several limitations in terms of toxicity assessment, increased variability, and population diversity. For example, rare but serious AEs may not be observed in small studies because of the low number of participants. Consequently, these trials may underestimate the risks associated with a treatment, leading to a different safety profile once the treatment is used in a larger population. Finally, smaller trials may not adequately represent the broader population, including variations in age, sex, ethnicity, and comorbidities. This lack of diversity can lead to skewed results, in which certain groups may experience different safety profiles that go unrecognized.

Copanlisib PK across the two dose levels evaluated and nivolumab PK (albeit limited sampling) were captured by their established population PK models (with some adjustment for nivolumab). Of note, the population PK-derived individual clearance values for copanlisib were consistent with historical data from the CHRONOS-1 monotherapy study (19). Similarly, nivolumab population PK–derived exposure parameters were consistent with those reported previously for nivolumab (40). These analyses support a lack of clinically relevant PK interaction for copanlisib and nivolumab in combination; this may be expected as copanlisib is eliminated via metabolism through hepatic cytochrome P450 3A enzymes (5), whereas disposition of nivolumab, a mAb, is expected to be mediated via typical antibody catabolism (10). No obvious copanlisib exposure influence was observed for treatment responses, which may be due to the limited number of responses reported in this study. Overall, PK results from this study support the combinability of copanlisib with nivolumab and the selection of copanlisib 60 mg as the RP2D to achieve established efficacious exposures.

It is noteworthy that the efficacy results reported in this article are limited by the small sample size and should be interpreted with caution. The ORR was 18.8% overall and approximately fourfold higher in the copanlisib 45-mg cohort (40.0%) than in the copanlisib 60-mg cohort (9.1%), possibly due to the smaller number of patients in the 45-mg cohort (responding tumor types included urothelial carcinoma of the bladder, choroidal melanoma, and oropharyngeal tongue cancer). The overall ORR falls within the range of that reported in previous monotherapy studies of copanlisib and nivolumab, in which an ORR ranging from 6% to 59% has been reported (6, 26–28, 41), supporting preclinical findings of the antitumor effect of these agents.

As recently discussed in a nonclinical biomarker analysis of copanlisib, pan-PI3K inhibition with copanlisib mitigated CD8^+^ Treg– and M2 TAM–mediated immune suppression, promoting antitumor responses (13). As this observed *in vivo* antitumor efficacy was partially due to immunomodulatory activity of copanlisib, we wanted to evaluate if baseline immune composition of patient tumors could be correlated with observed clinical efficacy following treatment. However, as the baseline data showed a low baseline infiltration of Tregs (*n* = 12; mean = 5.89% ± 6.09%) in the tumor microenvironment, immunomodulatory effects of copanlisib on the limited number of immune cells in the microenvironment may not have been sufficient to achieve the antitumor activity previously observed *in vivo*.

MDSCs, which can be characterized by CD11b^+^ CD33^+^ expression and divided into several subtypes based on phenotypic, morphologic, and functional heterogeneity, have emerged as major regulators of the immune response in cancer and are the subject of intensive research (42, 43). Although a number of studies published over the last few years have shown the immunomodulatory effects of PI3K inhibition, particularly the inhibition of Treg expansion and blockade of MDSC expansion, the mechanism of this immunomodulatory activity remains unclear (44–46). Therefore, we sought to assess changes in the immune environment following treatment with copanlisib via flow cytometry. The flow cytometry data reported herein supported an immunomodulatory effect of copanlisib as demonstrated by the maximum decrease in circulating MDSCs observed 2 days after treatment. These results support significant preclinical reductions in MDSC levels observed for other PI3K inhibitors, such as the first-in-class, orally administered PI3K-γ inhibitor eganelisib (47). Interestingly, a growing body of evidence has demonstrated a relationship between response to PD-L1 treatment and MDSC markers that have been linked with high immunosuppressive activity (48). This may suggest that the optimum dosing schedule of nivolumab is 24 to 48 hours after copanlisib, when a maximum decrease in MDSCs is observed. Indeed, in line with this finding, the lack of an additive effect observed for the combination compared with previously reported single-agent efficacy may be due to the need for further optimization of the dosing schedule. However, further evaluation is required as this is the first copanlisib clinical study to report these immunomodulatory changes in MDSCs, and the early stage of this study and the small number of patients limit the ability to provide definitive explanations at this time. A detailed systemic analysis could improve the understanding of these changes and optimize patient treatment.

As immunologic memory is a cardinal feature of adaptive immunity, we were interested in understanding the association between baseline levels of immune cells and treatment response in patients. We showed that lower baseline levels of CD8^+^ effector memory T cells, which confer immediate but not sustained effector function and produce cytotoxic proteins and cytokines (49, 50), seemed to correlate with better response. To further improve knowledge in this area, we investigated the role of cytokines that influence T-cell biology and showed that low levels of circulating IL-2 also seemed to correlate with improved tumor response. The role of IL-2 is well established in CD8^+^ T-cell biology as providing a signal to optimize effector T-cell generation and differentiation into memory cells (49, 51). As IL-2 is also known to induce expansion of Tregs and therefore favor immune suppression (51), we also evaluated the change in Treg levels using flow cytometry. Although no changes were observed in the Treg population after copanlisib treatment, the shared association of lower baseline levels of CD8^+^ effector memory cells and circulating IL-2 levels with improved tumor response suggests that reduced IL-2–dependent expansion of effector memory T cells may be predictive of patient outcomes. Of note, our biomarker results are limited by the small number of patients with biomarker data and should therefore be interpreted with caution.

Although there remains a large need for clinical biomarker samples that can effectively inform the disease landscape and potential treatment outcomes for patients with advanced solid tumors, we were largely unable to correlate patient responses with other biomarker signals examined. As this phase I study was open to all solid tumors, a key constraint for the performance of these analyses related to the limited number of samples collected in multiple tumor types. Additionally, although our use of archival tissue for these analyses was helpful from a patient burden standpoint and likely improved the screening failure rate, access to fresh paired biopsies is optimum for biomarker identification and may have potentially improved the quality and interpretability of the analyses presented here.

In conclusion, the RP2D of copanlisib administered in combination with nivolumab was established as 60 mg in patients with advanced solid tumors. No new safety concerns were identified with this treatment combination, and preliminary efficacy assessments indicated an antitumor effect, supporting further investigation of this treatment combination and the need for recent and future studies examining novel combinations of copanlisib and alternative ICI therapies (52–54).

## Supplementary Material

Table S1 Representativeness of study participants

Table S2 Criteria for defining dose-limiting toxicities

Table S3 Biomarker sampling time points

Figure S1 Prediction-corrected visual predictive checks of the final copanlisib population PK model in describing copanlisib PK in the present study

Figure S2 Prediction‐corrected visual predictive checks of the nivolumab population PK model in describing nivolumab PK in the present study

Figure S3 Distribution of individual copanlisib clearance values in the present study and in the Phase II copanlisib monotherapy study, CHRONOS-1

Figure S4 Distribution of individual copanlisib AUC[0–168]nd values across copanlisib dose levels

## Footnotes

1 On March 18, 2024, the FDA New Drug Application for copanlisib in adult patients with relapsed follicular lymphoma who have received ≥2 therapies was voluntarily withdrawn (24), and the clinical program for copanlisib has since been terminated.
