# Supplementary material for: Safety and Efficacy of Copanlisib in Combination with Nivolumab: A Phase Ib Study in Patients with Advanced Solid Tumors
Source: Cancer Res Commun. 2025 Mar 14;5(3):444–57. doi: 10.1158/2767-9764.CRC-24-0407 (PMC11907410; doi:10.1158/2767-9764.CRC-24-0407)
Supplement: Table S1 — Representativeness of study participants [file crc-24-0407_table_s1_suppst1.pdf]

Table S1. Representativeness of study participants

**Cancer type, subtype, stage, condition**	Histologically confirmed advanced solid tumors (bladder, choroidal melanoma, head and neck, kidney, non-small cell lung, oropharyngeal, small cell lung, squamous cell carcinoma, and squamous cell carcinoma of the tongue) where nivolumab is indicated<br>Bladder (25.0%) and oropharyngeal (18.8%) cancer were the most common cancer types in our study; considerations related to these two cancer types are further described below
**Considerations related to:**	
**Sex**	Age-adjusted incidence rates for bladder cancer and mouth/oral cancer are higher for men (9.3 and 5.8 per 100,000, respectively) than for women (2.4 and 2.3 per 100,000, respectively) (1,2)
**Age**	The median ages at diagnosis of bladder cancer and oropharyngeal cancer are 73 and 64 years, respectively (3,4)
**Race/ethnicity**	In the USA, White individuals are at the greatest risk of bladder and oropharyngeal cancer compared to other racial and ethnic groups (5,6)<br>Age-adjusted incidence for bladder cancer in males and females are 37.7 and 9.3 (White population), 20.0 and 5.1 (non-Hispanic American Indian/Alaska Native population), 18.9 and 5.8 (non-Hispanic Black population), 17.0 and 4.7 (Hispanic population), and 14.7 and 3.5 (non-Hispanic Asian/Pacific Islander population) per 100,000, respectively (3)<br>Age-adjusted incidence rates for oral cavity and pharynx cancer in males and females are 20.1 and 7.2 (White population), 18.0 and 5.8 (non-Hispanic American Indian/Alaska Native population), 12.7 and 4.9 (non-Hispanic Black population), 12.0 and 5.6 (non-Hispanic Asian/Pacific Islander population), and 10.2 and 4.5 (Hispanic population) per 100,000, respectively (4)
**Geography**	Globally, age-adjusted incidence rates for bladder cancer are highest in Western Europe (14.9 per 100,000), Central Europe (12.6 per 100,000), and North Africa and the Middle East (9.6 per 100,000) (7). Mortality from bladder cancer is also highest in these regions (7)<br>Globally, age-adjusted incidence rates for oropharyngeal cancer are highest in Western, Northern, and Central Europe (2.3-2.8 per 100,000), North America (2.4 per 100,000), and

	Australia and New Zealand (2.3 per 100,000) (8). Mortality from oropharyngeal cancer is highest in Central-East Europe, Western Europe, Melanesia, South-Central Asia, and the Caribbean (8)
**Other considerations**	Due to the confirmatory Phase III trial of copanlisib with standard immunochemotherapy (rituximab and bendamustine) in patients with relapsed lymphoma (CHRONOS-4) failing to reach its endpoint (9), the sponsor voluntarily withdrew the New Drug Applications for copanlisib from markets where single-agent copanlisib had been approved for relapsed follicular lymphoma (10)
**Overall representativeness of this study**	The age distribution of patients in our study (median age 65 years [range 37-89]) is similar to the age distribution of cases of bladder cancer and oropharyngeal cancer reported in national surveillance programs in the USA (3,4)
	The uneven distribution of males (75%) and females (25%) in our study reflects the higher incidence of bladder cancer and oropharyngeal cancer in males (1,2), suggesting fair representation of sexes in our study
	Our study was conducted across the USA and Canada only and is therefore not representative of the global bladder and oropharyngeal cancer population

References

1. World Cancer Research Fund International. Bladder cancer statistics. Available at: https://www.wcrf.org/cancer-trends/bladder-cancer-statistics/. Accessed August 9, 2024.

2. World Cancer Research Fund International. Mouth and oral cancer statistics. Available at: https://www.wcrf.org/cancer-trends/mouth-and-oral-cancer-statistics/#:~:text=Latest%20mouth%20and%20oral%20cancer,shown%20in%20the%20tables%20below. Accessed August 9, 2024.

3. National Cancer Institute. SEER cancer stat facts: bladder cancer. Available at: http://seer.cancer.gov/statfacts/html/urinb.html. Accessed August 12, 2024.

4. National Cancer Institute. SEER cancer stat facts: oral cavity and pharynx cancer. Available at: https://seer.cancer.gov/statfacts/html/oralcav.html. Accessed August 12, 2024.

5. American Cancer Society. Key statistics for oral cavity and oropharyngeal cancers. Available at: https://www.cancer.org/cancer/types/oral-cavity-and-oropharyngeal-cancer/about/key-statistics.html#:~:text=These%20cancers%20are%20more%20than,White%20people%20than%20Black%20people. Accessed August 12, 2024.

6. Rosiello G, Palumbo C, Deuker M, Stolzenbach LF, Martin T, Tian Z, et al. Racial differences in the distribution of bladder cancer metastases: a population-based analysis. Cent European J Urol 2020;73:407-15.

7. Safiri S, Kolahi AA, Naghavi M. Global, regional and national burden of bladder cancer and its attributable risk factors in 204 countries and territories, 1990-2019: a systematic analysis for the Global Burden of Disease study 2019. BMJ Glob Health 2021;6.

8. Lorenzoni V, Chaturvedi AK, Vignat J, Laversanne M, Bray F, Vaccarella S. The current burden of oropharyngeal cancer: a global assessment based on GLO1BOCAN 2020. Cancer Epidemiol Biomarkers Prev 2022;31:2054-62.

9. Zinzani PLL, Wang H, Feng J, Kim TM, Tao R, Zhang H, et al. CHRONOS-4: Phase 3 study of copanlisib plus rituximab-based immunochemotherapy in relapsed indolent B-cell lymphoma. Blood Adv 2024; Epub ahead of print. doi: 10.1182/bloodadvances.2024013236.

10. US Food and Drug Administration. Bayer HealthCare Pharmaceuticals Inc.; withdrawal of approval of New Drug Application for ALIQOPA (copanlisib) for injection, 60 milligrams per vial. Available at: https://www.regulations.gov/document/FDA-2024-N-1180-0001. Accessed June 18, 2024.
