# Supplementary material for: Safety and Efficacy of Copanlisib in Combination with Nivolumab: A Phase Ib Study in Patients with Advanced Solid Tumors
Source: Cancer Res Commun. 2025 Mar 14;5(3):444–57. doi: 10.1158/2767-9764.CRC-24-0407 (PMC11907410; doi:10.1158/2767-9764.CRC-24-0407)
Supplement: Table S2 — Criteria for defining dose-limiting toxicities [file crc-24-0407_table_s2_suppst2.pdf]

Table S2. Criteria for defining dose-limiting toxicities

Toxicology	Criteria[a,b]
**Hematology**	Grade ≥4 neutropenia for ≥7 consecutive days<br>Grade ≥4 thrombocytopenia<br>Grade ≥3 thrombocytopenia with serious bleeding<br>Grade ≥3 anemia<br>Febrile neutropenia (ANC <1.0×10^9/L with a single temperature >38.3°C or a sustained temperature of ≥38°C for more than 1 hour)
**Gastrointestinal**	Grade ≥4 diarrhea<br>Grade ≥3 diarrhea which is not responding to anti-diarrheal treatment<br>Grade ≥3 nausea or grade ≥3 vomiting not responding to optimal anti-emetic therapy
**Hepatobiliary**	Grade ≥3 AST/ALT increase with elevation of serum total bilirubin to >2×ULN or >50% increase from baseline, without initial findings of cholestasis (elevated serum ALP)
**Renal**	Grade ≥4 serum creatinine increase<br>Grade ≥3 serum creatinine increase not corrected after intravenous fluids
**Non-hematologic events**	Grade ≥3, except for the exclusions noted below
**Exceptions to DLT criteria**	Grade ≥3 laboratory abnormalities that are responsive to oral supplementation or deemed by the investigator to be clinically insignificant<br>Grade 3 infusion-related hyperglycemia lasting ≤7 days<br>Grade 3 infusion-related hypertension<br>Grade 3 hypothyroidism<br>Grade 3 fatigue lasting ≤7 days

[a]DLTs were defined as any of these prespecified events that occurred during cycle 1 or 2 considered related to the study treatment; [b]Common Terminology Criteria for Adverse Events version 5.0 was used for all grading

ALP, alkaline phosphatase; ALT, alanine aminotransferase; ANC, absolute neutrophil count; AST, aspartate aminotransferase; DLT, dose-limiting toxicity; ULN, upper limit of normal
