# Supplementary material for: Safety and Efficacy of Copanlisib in Combination with Nivolumab: A Phase Ib Study in Patients with Advanced Solid Tumors
Source: Cancer Res Commun. 2025 Mar 14;5(3):444–57. doi: 10.1158/2767-9764.CRC-24-0407 (PMC11907410; doi:10.1158/2767-9764.CRC-24-0407)
Supplement: Table S3 — Biomarker sampling time points [file crc-24-0407_table_s3_suppst3.pdf]

Table S3. Biomarker sampling time points

Cycle	Day	Time point relative to study intervention infusion	Whole blood for immune cell profiling	Biomarker plasma
Screening	−7 to −1		X	X
1	1	Before infusion	X	X
1	2	24 hours after first infusion	X	X
1	3	48 hours after first infusion	X	X
1	8	Before cycle 1, day 8	X	X
1	15	Before cycle 1, day 15	X	X
1	22		X	X
2	1	Before infusion	X	X
2	8	Before infusion	X	X
2	15	Before infusion	X	X
3	8	Before infusion	X	X
4	8	Before infusion	X	X
Subsequent even cycles (cycle 6, cycle 8, etc.)	8	Before infusion	X	X
End of treatment			X	X
