# Supplementary material for: Safety and Efficacy of Copanlisib in Combination with Nivolumab: A Phase Ib Study in Patients with Advanced Solid Tumors
Source: Cancer Res Commun. 2025 Mar 14;5(3):444–57. doi: 10.1158/2767-9764.CRC-24-0407 (PMC11907410; doi:10.1158/2767-9764.CRC-24-0407)
Supplement: Figure S1 — Prediction-corrected visual predictive checks of the final copanlisib population PK model in describing copanlisib PK in the present study [file crc-24-0407_figure_s1_suppsf1.pdf]

Figure S1. Prediction-corrected visual predictive checks of the final copanlisib population PK model in describing copanlisib PK in the present study

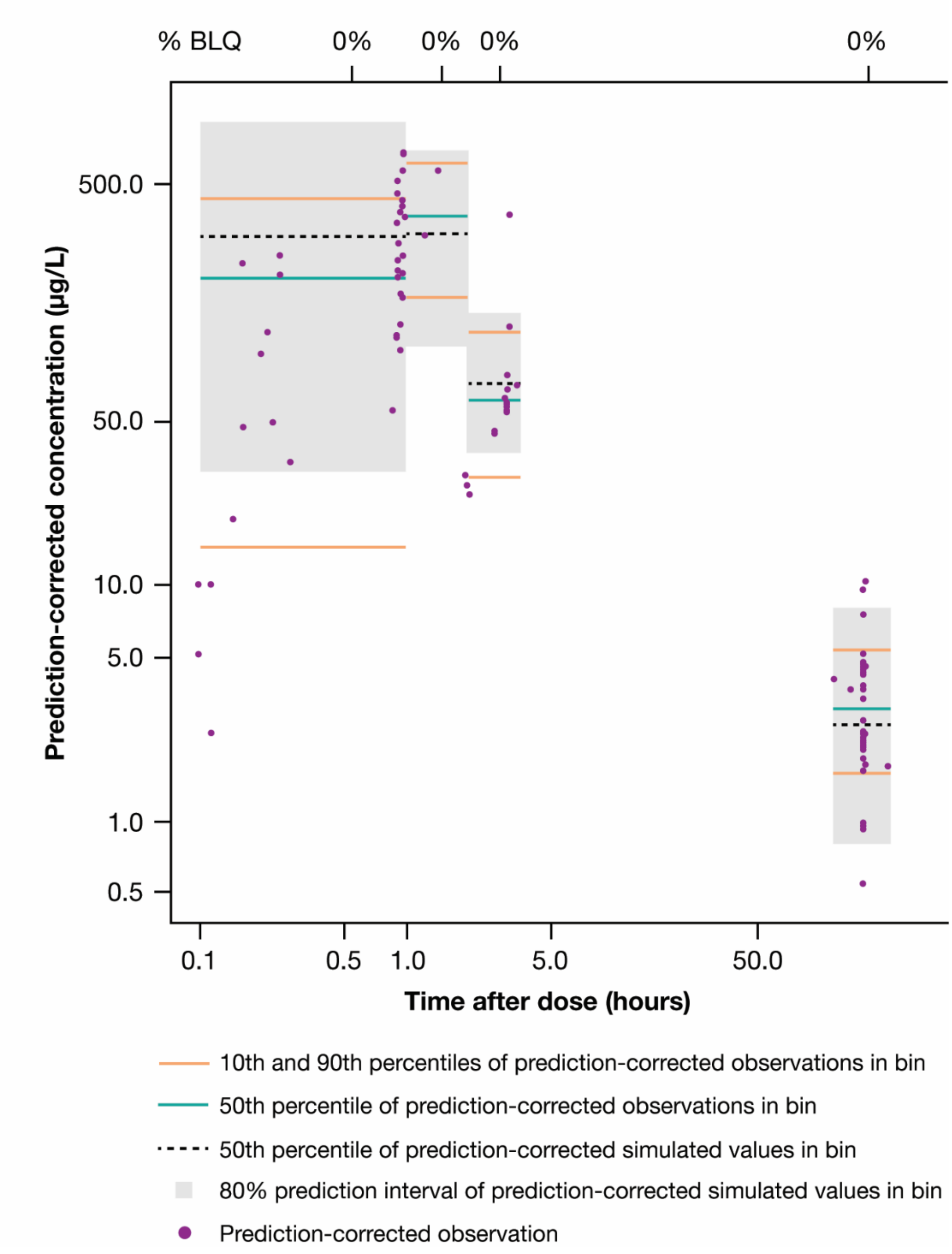

Orange lines and shaded regions correspond to model simulations and green lines correspond to observed data. Purple circles represent prediction-corrected observations that are greater than the lower limit of quantification. Green horizontal

lines represent the 50th percentiles of prediction-corrected observations in the bin. Orange horizontal lines represent the 10th and 90th percentiles of prediction-corrected observations in the bin. Black dashed horizontal lines represent the 50th percentile of prediction-corrected simulated values in the bin. The gray-shaded areas represent the 80% prediction interval of prediction-corrected simulated values in the bin. The numbers along the top of the plot represent the percentage of observations in the bin that are less than the lower limit of quantification

BLQ, below the limit of quantification; PK, pharmacokinetics
