# Supplementary material for: Safety and Efficacy of Copanlisib in Combination with Nivolumab: A Phase Ib Study in Patients with Advanced Solid Tumors
Source: Cancer Res Commun. 2025 Mar 14;5(3):444–57. doi: 10.1158/2767-9764.CRC-24-0407 (PMC11907410; doi:10.1158/2767-9764.CRC-24-0407)
Supplement: Figure S2 — Prediction‐corrected visual predictive checks of the nivolumab population PK model in describing nivolumab PK in the present study [file crc-24-0407_figure_s2_suppsf2.pdf]

**Figure S2.** Prediction-corrected visual predictive checks of the nivolumab population PK model in describing nivolumab PK in the present study

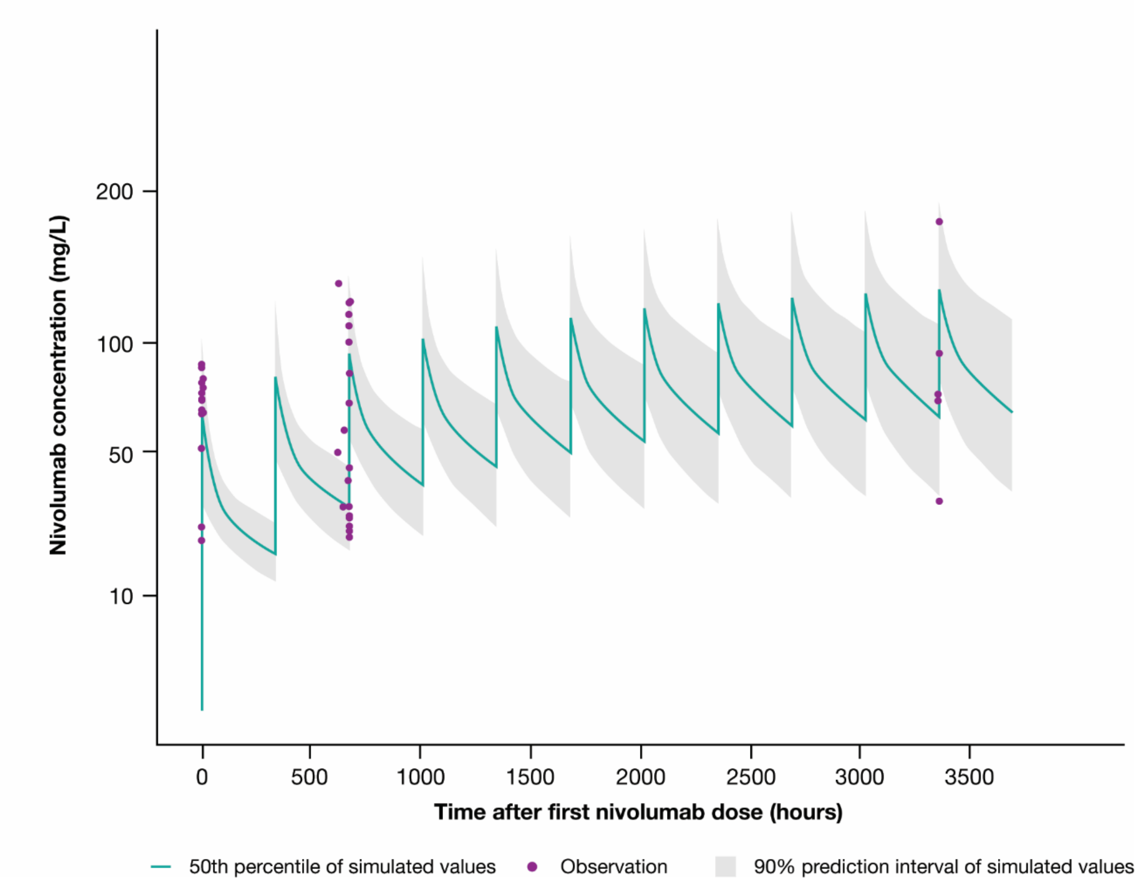


The gray-shaded regions represent the 90% prediction interval resulting from a simulation of 1000 virtual patients according to the published population PK model. Green lines represent the median simulated values. Residual error and inter-individual variability in residual error were included in the simulation. Purple circles represent the observed nivolumab concentrations

PK, pharmacokinetics
