# Supplementary material for: Safety and Efficacy of Copanlisib in Combination with Nivolumab: A Phase Ib Study in Patients with Advanced Solid Tumors
Source: Cancer Res Commun. 2025 Mar 14;5(3):444–57. doi: 10.1158/2767-9764.CRC-24-0407 (PMC11907410; doi:10.1158/2767-9764.CRC-24-0407)
Supplement: Figure S3 — Distribution of individual copanlisib clearance values in the present study and in the Phase II copanlisib monotherapy study, CHRONOS-1 [file crc-24-0407_figure_s3_suppsf3.pdf]

Figure S3. Distribution of individual copanlisib clearance values in the present study and in the Phase II copanlisib monotherapy study, CHRONOS-1

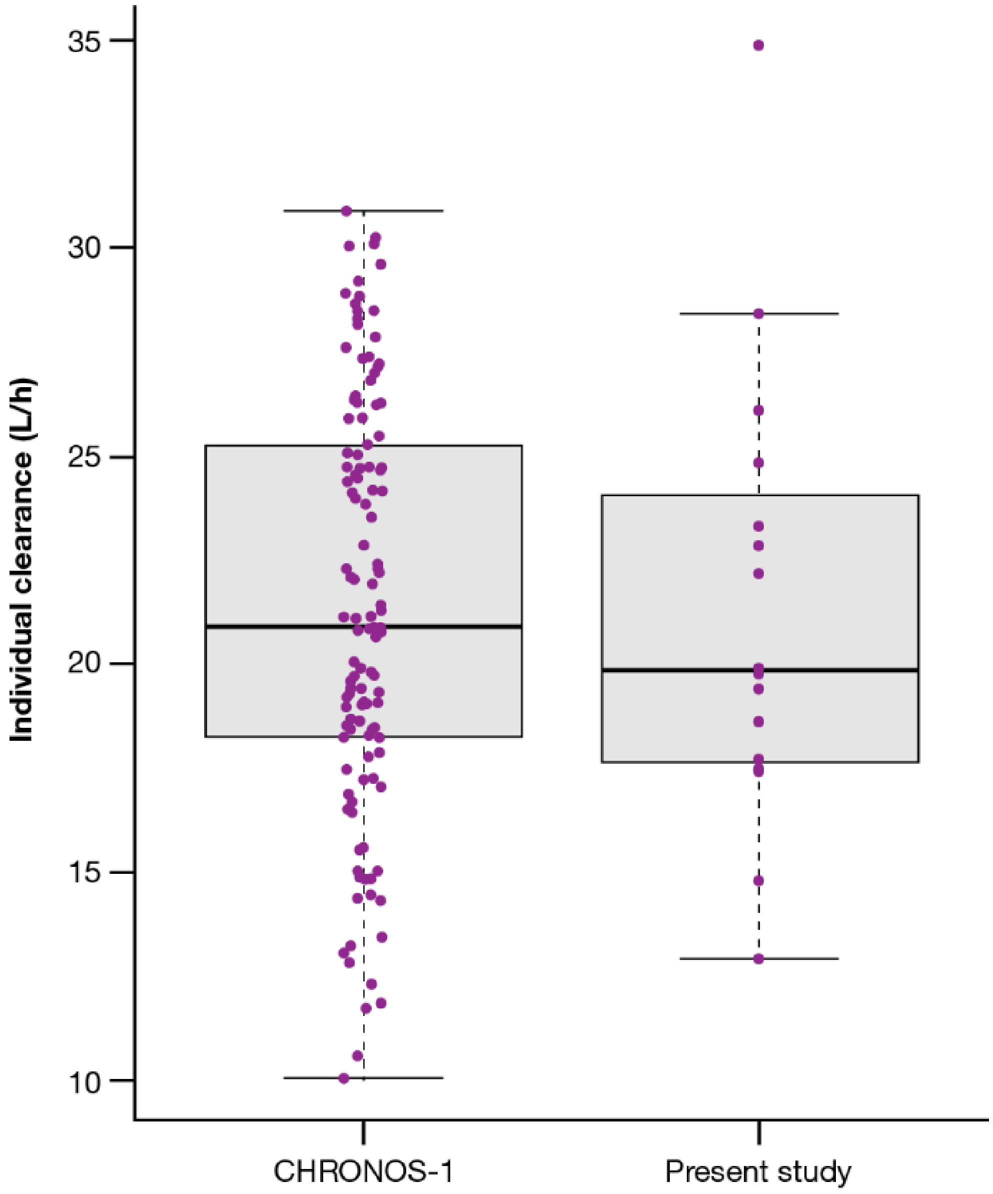

The boxes represent the interquartile ranges of exposure variables, and the thick black lines represent the median values. The ends of the whiskers are at the largest value that is within 1.5 times the interquartile range from the median. The circles represent individual values
