# Supplementary material for: Safety and Efficacy of Copanlisib in Combination with Nivolumab: A Phase Ib Study in Patients with Advanced Solid Tumors
Source: Cancer Res Commun. 2025 Mar 14;5(3):444–57. doi: 10.1158/2767-9764.CRC-24-0407 (PMC11907410; doi:10.1158/2767-9764.CRC-24-0407)
Supplement: Figure S4 — Distribution of individual copanlisib AUC[0–168]nd values across copanlisib dose levels [file crc-24-0407_figure_s4_suppsf4.pdf]

Figure S4. Distribution of individual copanlisib $AUC_{[0\text{-}168]nd}$ values across copanlisib dose levels

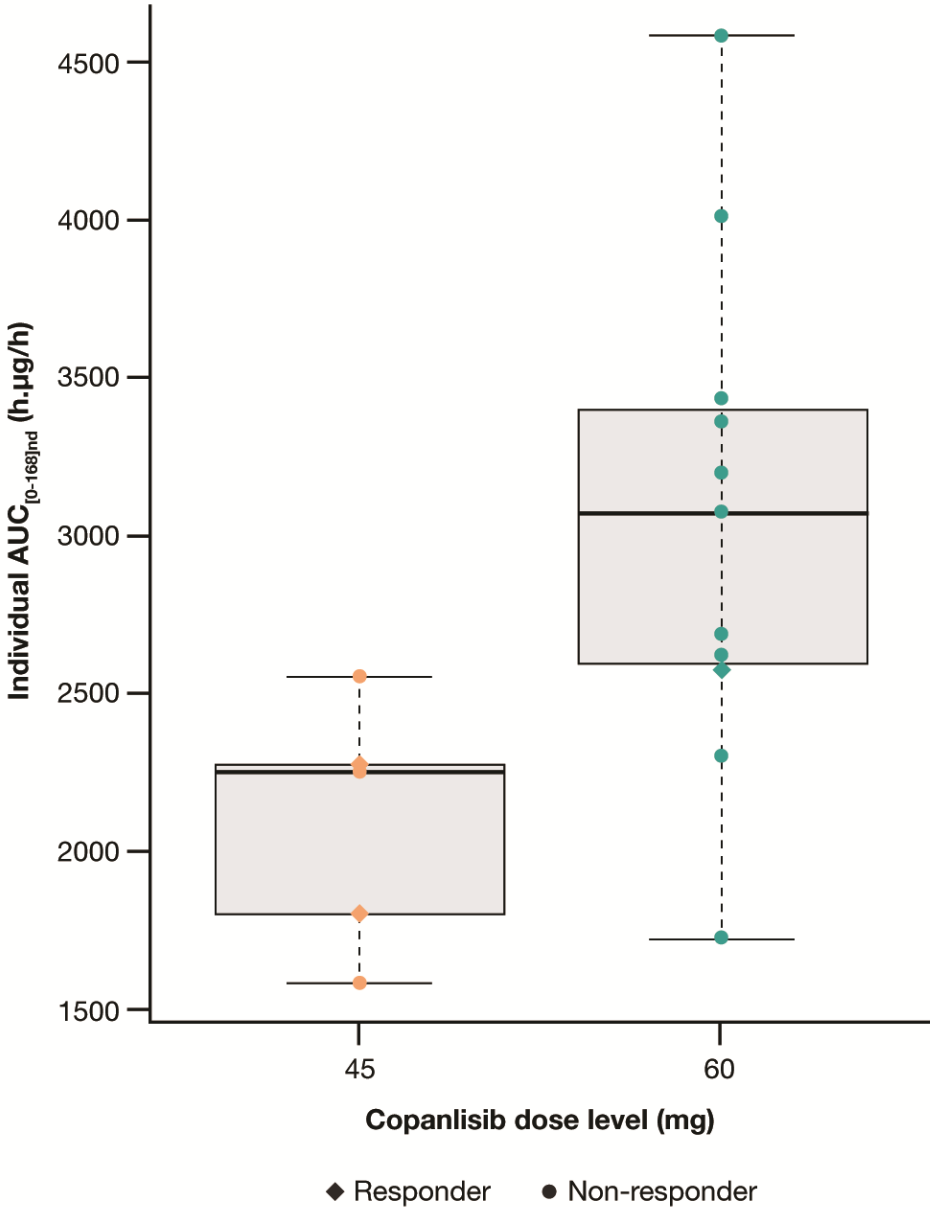

The boxes represent the interquartile ranges of the exposure variables, and the thick black lines represent the median values. The ends of the whiskers are at the largest value that is within 1.5 times the interquartile range from the median. The circles and diamonds represent individual values

$AUC_{[0\text{-}168]nd}$, area under the curve from 0 to 168 hours following nominal dosing
